# Clinical and laboratorial profiles of dengue virus infection in kidney transplant recipients: Report of a single center

**DOI:** 10.1371/journal.pone.0219117

**Published:** 2019-10-30

**Authors:** Ida Maria Maximina Fernandes-Charpiot, Cassia Fernanda Estofolete, Heloisa Cristina Caldas, Gabriela Rodrigues de Souza, Rita de Cássia Martins Alves da Silva, Maria Alice Sperto Ferreira Baptista, Mauricio Lacerda Nogueira, Mario Abbud-Filho

**Affiliations:** 1 Kidney Transplant Unit, Faculty of Medicine of São José do Rio Preto, FAMERP—Hospital de Base São José do Rio Preto, São José do Rio Preto, Brazil; 2 Laboratory of Research in Virology, Faculty of Medicine of São José do Rio Preto, FAMERP, São José do Rio Preto, Brazil; 3 Instituto de Urology e Nefrologia, São José do Rio Preto, Brazil; Institut Pasteur of Shanghai Chinese Academy of Sciences, CHINA

## Abstract

Dengue infection (DI) is the most important arboviral infection in the world. The majority of immunocompetent patients will have asymptomatic or mild infections, but the degree of dengue severity in kidney transplant recipients (KTx) is unknown. In this study, we report the clinical profile and outcomes of 39 dengue cases in KTx. From a total of 1,186 KTx outpatients in follow-up we reviewed clinical and laboratory records of 60 (5%) patients admitted with suspected DI initially screened by NS-1, IgM, and when possible, multiplex nested PCR. The prevalence of DI in KTx was 3% (39/1,118), with symptoms leading to hospital admission being fever, myalgia, malaise, and headache. Laboratory tests showed leucopenia, thrombocytopenia, and liver enzyme elevation. DI was confirmed by positivity of NS-1 (33%), IgM (69%), and/or RT-PCR (59%). Twenty-three patients (59%) had dengue with warning signs, and 15% had severe dengue, 2 of them with a fatal course. Acute graft dysfunction occurred in 59% (mean nadir serum creatinine: 2.9 ± 2.6mg/dL), 4 of them requiring dialysis. CMV coinfection diagnosed in 19% of the cases and patients was associated with worse clinical presentation. Our results suggest that KTx with DI presented initial physical and laboratorial profile similar to the general population. However, DI in KTx seems to have a higher risk for graft dysfunction, severe dengue, and death. Because CMV coinfection aggravates the DI clinical presentation and recovery, it must be evaluated in all cases.

## Introduction

Dengue virus (DENV), an arbovirus transmitted by the *Aedes* mosquito, is the causative agent of Dengue infection (DI) and is the major cause of morbidity and mortality in many endemic Asian and South American countries [1–3]. DENV is responsible for major urban outbreaks that are usually associated with the introduction of a new serotype [4–6]. In 2016, 1,483,623 cases of DI were reported in Brazil, of which 919 were characterized as severe dengue, and 9,153 had DI with warning signs. In addition, in the last two years, 842 deaths were associated with dengue in Brazil [7]. Our region is a DENV surveillance and control area due to the endemic circulation of different serotypes [8–11] and, during the period from 2015 to 2016, our city faced a dengue epidemic, with 21,839 confirmed and 16,291 dengue-probable cases [12,13].

All four dengue virus serotypes (DENV-1 to -4) can cause severe infection which sometimes leads to death, but the majority of patients will have asymptomatic infections or mild symptoms, characterized by influenza-like disease [2,3,14]. Other clinical classifications of the disease, such as dengue with warning signs (D+WS), a more symptomatic form of the disease, and severe dengue (SD), characterized by severe organ impairment which may lead to death, have increased their incidence [15–17].

In recent decades, the growing number of organ transplant recipients in developing countries, living in or traveling to an endemic area of dengue, is at risk of developing this infection [18].

Additionally, kidney transplant recipients (KTx), DI may be associated with other viral infections, such as cytomegalovirus (CMV), increasing the risk for the recipients after renal transplantation (Tx) [19]. Due to a very limited amount of data on the consequences of DI and controversial results in KTx recipients, in the present study we aimed to evaluate the clinical profile of 39 patients diagnosed with DI. Because CMV remains one of the most important viruses affecting KTx [20] and it may be associated with DI in 5% [18] to 66% [21] of these cases, it is not clear whether the CMV coinfection may modify the outcome of DI as it usually does in other viral coinfections [21]. Therefore, in addition to the DI clinical profile, here we also sought to characterize the possible effects of the DI coinfection with CMV in a single center in different outbreak periods.

## Material and methods

We reviewed the records of 1,186 KTx outpatients in follow-up from January of 2007 to December of 2016 at our service. During this period, 60 (5%) KTx with suspected DI were admitted to a public university hospital, in the northwest region of São Paulo State, and 39 (3.3%) of them had a confirmed diagnosis of DI. This work is part of an arbovirus surveillance program approved by the Ethics Committee in Research of the Faculty of Medicine of São José do Rio Preto–FAMERP (ethics application no. 15461513.5.0000.5415/2013) with waiver of informed consent as this was a retrospective study and the data were analyzed anonymously. The study was conducted in accordance with the Declaration of Helsinki.

Retrospective review of demographic data, acute rejection surveillance from transplant to 6 months after DI, serum creatinine, immunosuppressive regimens, main clinical manifestations, time elapsed between transplantation and diagnosis, and mortality rate up to 30 days after DI and for the entire follow-up were collected. Hemoconcentration was characterized by a 20% increase in the basal hematocrit (Ht) or Ht > 40% in women and Ht > 45% in men [22]. Serum creatinine measurements were analyzed 3 times: at the time of DI diagnosis, 30 days after discharge, and 6 months after DI. The nadir of serum creatinine was monitored during patient hospitalization.

All patients were initially screened for DENV by NS-1 and/or IgM (Alere S.A.). The same samples, when possible, were also screened for DENV using multiplex nested PCR [22], and CHIKV and ZIKV using RT-qPCR [23,24].

The serum was separated and the viral RNA was extracted from 140 μL of each serum sample using the QIAamp Viral RNA Mini Kit (QIAGEN, Germany) according to the manufacturer´s instructions. The RNA was analyzed using qRT-PCR in the cases of ZIKV [24] and CHIKV [23], and using RT-PCR in the case of DENV [25]. There was no genetic sequencing of viral lineages. This methodology was validated in previous publications [23–25].

For 19 cases in which DI was not confirmed, the following diagnoses were made: cytomegalovirus infection (n = 5), urinary infection (n = 4), gastrointestinal infection (n = 3), viral pneumonia (n = 2), and 1 case of biliary infection. In 4 cases, we could not identify any pathogen. Additionally, 2 cases of Zika, not associated with DI, were diagnosed during the 2015 outbreak [26].

Diagnosis of DI was confirmed in the laboratory by using at least one of these serum tests: Non-structural 1 antigen (NS-1) detection (NS-1 enzyme-linked immunosorbent assay), detection of immunoglobulin M (IgM) (IgM anti-dengue serology), and/or RT-PCR (reverse-transcriptase polymerase chain reaction) [8, 27]. Diagnosis of CMV infection was made using quantitative CMV PCR (Q-CMV real-time complete kit (Nanogen Advanced Diagnostic S.r.L. Italy or COBAS AmpliPrep/COBAS TaqMan CMV. Clinical and laboratory data were organized in tables and analyzed according to the 2009 World Health Organization (WHO) Dengue Classification [15] (Table 1).

**Table 1:** Demographic characteristics of 39 transplant recipients at initial diagnosis of dengue infection.

Baseline characteristics	Mean ± SD or n (%) n = 39
Age (years)	50 ± 14
Female gender	22 (56%)
Ethnicity (Caucasian)	30 (77%)
*Renal disease*	
Polycystic kidney disease	8 (21%)
Hypertension	6 (15%)
Glomerulopathy	6 (15%)
Diabetes mellitus	4 (10%)
Unknown	15 (39%)
*Donor*	
Deceased	22 (56%)
Living	17 (44%)
*Induction therapy*	
Basiliximab	18 (46%)
Thymoglobulin	04 (10%)
None	17 (44%)
*Immunosuppression*	
Calcineurin inhibitors	27 (70%)
mTOR inhibitors	15 (39%)
Micophenolic acid	22 (56%)
Steroid	39 (100%)
Acute rejection prior to DI diagnosis	8 (20%)
CMV infection prior to DI diagnosis	12 (31%)
Time since transplant (months)	65 ± 62
Hospitalization required	29 (74%)
Hospitalization time (days)	7 ± 6
Time onset symptoms (days)	5 ± 4
Follow-up time after DI (months)	28 ± 24

Data are presented as absolute numbers, means ± standard deviations, medians or percentages. The Mann-Whitney U and Kruskal-Wallis tests were used to determine statistical significance for continuous variables, and chi-square or Fisher's exact test for categorical variables. All analyses were performed using Stats Direct version 3.0 (Stats Direct Ltd.). The statistical significance level established was a p value < 0.05.

### Ethical approval

This work is part of an arbovirus surveillance program approved by the Ethics Committee in Research of the Faculty of Medicine of São José do Rio Preto–FAMERP (ethics application no. 15461513.5.0000.5415/2013) with waiver of informed consent as this was a retrospective study and the data were analyzed anonymously. The study was conducted in accordance with the Declaration of Helsinki.

## Results

Demographic characteristics, along with the most common initial clinical symptoms and laboratorial findings of patients are shown in Tables 1 and 2. Thirty-nine cases (39/1,186 (3.3%) KTx in follow-up) of DI were confirmed, the majority during the outbreaks of 2013 (8/39; 21%), 2015 (15/39; 39%), and 2016 (9/39; 23%) (Table 3).

**Table 2:** Clinical Characteristics of Dengue Infection in KTx at Admission.

Characteristics Total n = 39		
Clinical findings (n/39)	n (%)	Mean ± SD
Fever	34 (87)	-
Myalgia	32 (82)	-
Malaise	27 (69)	-
Headache	24 (62)	-
Nauseas	18 (46)	-
Anorexia	18 (46)	-
Diarrhea	15 (39)	-
Arthralgia	9 (23)	-
Rash	6 (15)	-
**Coinfection**		-
Cytomegalovirus	6 (19)†	-
Hepatitis (B/C)	none	-
Laboratory findings*		
Hemoconcentration	3 (7.6)	-
Leukopenia	26 (67)	3,481 ± 2,010/mm^3^
Thrombocytopenia	29 (74)	117,665 ± 90,943/mm^3^
Increased AST*	21 (65)†	75 ± 59 UI/l
Increased ALT*	19 (59)†	71 ± 87 UI/l
Increased GGT*	22 (78)†	168 ± 240 UI/l
Hemoglobin (mg/dL)	-	12 ± 2
Hematocrit (%)	-	38 ± 5.7
Creatinine (mg/dL)	-	2.9 ± 2.6
**DI with warning signs (D+WS)**	23 (59)	-
Persistent vomiting	7 (57)	-
Abdominal pain	9 (39)	-
Hypotension	8 (35)	-
Bleeding	7 (30)	-
Clinical fluid accumulation (ascites)	2 (9)	-
Lethargy, restlessness	1 (4)	-
**Severe dengue**	6 (15)	-
Severe plasma leakage leading to shock	1 (2.5)	-
Severe bleeding (hemorrhagic stroke)	1 (2.5)	-
Severe organ involvement**	4 (10)	-
**Renal function**		
Mean serum creatinine (mg/dL)					
Last baseline	-					1.7 ± 0.8					
Nadir	-				2.9 ± 2.6
Day 30 after discharged	-				1.5 ± 0.7
Day 6 months after DI	-				1.6 ± 0.8
Acute graft dysfunction	23 (59)	-
Required renal replacement therapy	4 (17)	-
Renal function after 30 days		
Completely recovery	16 (70)	-
Partial recovery	4 (17)	-
**Outcomes**		
Graft loss	1 (3)	-
Death***	2 (5)	-

* AST: aspartate aminotransferase, ALT: alanine aminotransferase, GGT: gamma-glutamyltransferase. Reference values: Hb: 12–17 mg/dl; Ht: 40–55%; Leukocytes: 4,000–11,000/mm^3^; platelets: 150,000–300,000/mm^3^; Cr: 0.7–1,2 mg/dl; AST: 40 UI/l; ALT: 41 UI/l; GGT: 1–60 UI/l.

** Severe organ involvement: pancreas (n = 1, acute pancreatitis), kidney (n = 1, graft loss), and central nervous system (n = 2, encephalitis)

*** Death causes: hemorrhagic stroke (1), shock (1)

† Cytomegalovirus: n = 31; AST and ALT: n = 32; GGT: n = 28

**Table 3:** Confirmatory diagnosis of dengue in each KTx case.

	Confirmatory Laboratory Method				
Patient	NS1 detection	IgM—ELISA	RT-PCR	CSF	Year
	13/18 (72%)	27/32 (84%)	22/28 (79%)	2/2 (100%)	
1	ND	ND	**Serotype 3**	ND	2007
2	ND	ND	**Serotype 3**	ND	2007
3	ND	**Positive**	**Serotype 1**	ND	2010
4	**Positive**	Negative	**Serotype 2**	ND	2010
5	ND	**Positive**	ND	ND	2010
6	**Positive**	**Positive**	**Serotype 1**	ND	2011
7	ND	**Positive**	**Serotype 1**	ND	2013
8	**Positive**	**Positive**	Not amplified	ND	2013
9	**Positive**	**Positive**	**Serotype 1**	ND	2013
10	ND	**Positive**	ND	ND	2013
11	ND	**Positive**	Negative	ND	2013
12	**Positive**	ND	ND	ND	2013
13	ND	Inconclusive	**Serotype 1**	ND	2013
14	Negative	Negative	**Serotype 1**	ND	2013
15	**Positive**	**Positive**	**Serotype 1**	ND	2014
16	ND	Negative	**Serotype 1**	ND	2015
17	ND	**Positive**	**Serotype 1**	ND	2015
18	**Positive**	**Positive**	ND	ND	2015
19	**Positive**	ND	**Serotype 1**	ND	2015
20	ND	**Positive**	**Serotype 1**	ND	2015
21	Negative	**Positive**	**Serotype 1**	ND	2015
22	Negative	**Positive**	Negative	ND	2015
23	ND	**Positive**	Negative	ND	2015
24	**Positive**	Negative	**Serotype 1**	ND	2015
25	ND	ND	**Serotype 1**	ND	2015
26	Negative	**Positive**	**Serotype 1**	ND	2015
27	ND	**Positive**	Negative	ND	2015
28	ND	**Positive**	ND	ND	2015
29	ND	**Positive**	ND	ND	2015
30	Negative	**Positive**	Negative	**Serotype 1**	2015
31	ND	**Positive**	ND	ND	2016
32	**Positive**	**Positive**	ND	ND	2016
33	ND	**Positive**	ND	ND	2016
34	**Positive**	ND	**Serotype 2**	ND	2016
35	**Positive**	ND	**Serotype 2**	ND	2016
36	**Positive**	**Positive**	**Serotype 1**	ND	2016
37	ND	**Positive**	**Serotype 2**	ND	2016
38	ND	**Positive**	ND	ND	2016
39	ND	**Positive**	ND	Positive**	2016

ND: not done; CSF: cerebrospinal fluid

** IgM serology (ELISA)

Hospitalization was required in 74% of the cases, and the hospitalization time ranged from 2 to 28 days (average: 7 ± 6 days). Average time between the onset of symptoms and patient hospital admission was 5 ± 4 days (ranging from 1–20 days) and occurred 65 ± 62 months after transplantation (ranging from 1–210 months). Twenty-three (59%) were diagnosed as D+WS, and 6 of them (15%) developed SD.

Induction therapy was given in 56% of the cases with anti-lymphocyte antibodies (4/39) and basiliximab (18/39). Maintenance immunosuppression at the time of disease was a combination of calcineurin inhibitors, acid mycophenolic, and/or mTOR inhibitor with prednisone (Table 1). Acute rejection (AR) rate was 20% (8/39) after transplant and none had AR in the 6 months prior to DI. CMV infection rate was 31% (12/39) after transplant and 5% (2/39) had been treated for CMV infection/ disease in the 6 months prior to DI.

Adjustments in the immunosuppression (ISS) were necessary in 12 of 39 cases due to leukopenia, diarrhea, or high tacrolimus levels. In 8 (67%) of these 12 cases, the diagnosis was of SD, and 2 cases were classified as D+WS. All 12 patients with ISS changes (group w/) required hospitalization (vs. 63% without ISS changes (group w/o); p = 0.01). These patients also needed longer hospitalization periods (w/ = 9.8 ± 6.3 days vs. w/o = 5.2 ± 6 days; p = 0.008). Acute graft dysfunction occurred in 92% and 44% of groups w/ and w/o ISS, respectively; p 0.01. Nadir of serum creatinine was: w/ = 4.5 ± 3.9 mg/dL vs. w/o = 2.1 ± 1.2 mg/dL (p = 0.01); 33% required dialysis (vs. none in w/o ISS group; p = 0.009). CMV coinfection was present in 25% and 11% in w/ and w/o ISS group, respectively (NS). Death occurred only in the group w/ ISS changes (17%; NS) (S1 Table).

Table 3 shows that the diagnosis of DI was performed by at least one of the following tests: NS-1 detection (33%), IgM serology (69%), and/or RT-PCR (56%). Four (10%) of 39 KTx had all 3 tests positive, 17 (44%) had 2 tests positive and 18 (46%) had one test positive (Table 3). NS-1 detection was positive in 13/18 (72%), IgM serology in 27/32 (84%), and RT-PCR in 22/28 (87%) of the cases tested. Dengue serotypes were identified in 82% of the cases: virus 1 (DENV-1) was the main identified serotype (17/23; 74%), followed by DENV-2 (4/23; 17%), and DENV3 (2/23; 9%). One of the patients had DENV-1 serotype identified simultaneously by RT-PCR in the cerebrospinal fluid (CSF) and by IgM serology (negative blood RT-PCR and NS-1 detection). Another KTx had a positive CSF immunoglobulin G (IgG) serology test (Enzyme-Linked Immunosorbent Assay—ELISA, Euroimmun) associated with positive blood IgM serology. Only 1 patient- presented with negative serological tests (NS -1 and IgM serology) and positive RT-PCR (Table 3).

After day 5 of illness, 100% of patients tested had positive IgM serology, and 75% had RT-PCR (from day 6 to 15 of illness), but the NS-1 and PCR were superior to IgM serology when patients were admitted and tested before day 5 of illness (80% positive). Additionally, NS-1 results were known faster than PCR results. From 10 patients that underwent all 3 tests, 6 (60%) had positive NS-1, and 4 (40%) had positve IgM serology before day 5 of illness, and 100% had positive RT-PCR independent of the time of illness.

Acute graft dysfunction, characterized by a 25% increase in serum creatinine elevation from the baseline, occurred in 59% of the cases (mean creatinine from 1.7 ± 0.8 mg/dL to 2.9 ± 2.6 mg/dL), and 4/23 patients required dialysis. After 30 days, the great majority (16/23) of KTx had their serum creatinine returned to basal levels, and only 4 of them had creatinine levels partly returned to their respective baseline. Three patients lost their grafts; one patient needed dialysis and renal function did not recover after DI resolution, and two patients died undergoing dialysis from complications of DI (Table 2). After 28 ± 24 months, 31/39 of the patients were alive, and the mean serum creatinine was 1.6 ± 0.6mg/dL.

Twenty-three KTx were diagnosed with D+WS and presented with persistent vomiting, abdominal pain, bleeding, and ascites. Six of D+WS patients fulfilled the criteria for severe dengue (SD), and 4 of these were characterized with severe organ involvement including pancreas (acute pancreatitis followed by diabetes), kidney (acute graft dysfunction followed by graft loss), and central nervous system (two encephalitis, one followed by paraparesis). The 2 remaining SD had fatal course, one due to severe bleeding (hemorrhagic stroke) followed by brain death and the other had clinical fluid accumulation followed by shock (Table 2).

Coinfection with cytomegalovirus was present in 19% of the 31 cases in which the CMV-PCR test was performed (Table 4 shows the demographics of the two subgroups). The subgroup that had coinfection with CMV viremia contracted DI in a shorter time after transplant, had a longer hospitalization time, worse thrombocytopenia, higher liver enzyme (GGT) levels, and a higher rate of late death not related to DI and not related to CMV. None of the 39 KTx had co-infection with hepatitis B or C. No significant differences were observed among the subgroups under different immunosuppression therapy.

**Table 4:** Dengue infection in KTx subgroup which developed CMV viremia compared with KTx subgroup with PCR negative.

	PCR CMV + (n = 6) Mean ± SD or n (%)	PCR CMV—(n = 25) Mean ± SD or n (%)	p-value
Age (years)	54 ± 12	51 ± 14	0.63
Time since transplant (months)	17 ± 33	65 ± 64	0.01
Time onset symptoms (days)	7 ± 5	4 ± 4	0.1
Hospitalization time (days)	15 ± 9	6 ± 5	0.003
DI with warning signs	5 (83%)	14 (56%)	0.3
Severe dengue	1 (17%)	4 (16%)	1
Viral load of the CMV (UI)*	236–2,366	negative to < 29	-
Hemoglobin (mg/dl) ^#^	12 ± 3	12 ± 2	1
Leukocytes (cells/mm^#^	2 ± 57	3.5 ± 2	0.08
Platelets (cells/mm^3^)^#^	42.6 ± 40.5	120.3 ± 92.6	0.005
AST (UI/l)^#^	111 ± 79	72 ± 64	0.16
ALT (ui/l)^#^	115 ± 116	67 ± 86	0.3
Alkaline phosphatase (UI/l)^#^	192 ± 189	112 ± 107	0.2
Gamma-glutamyltransferase (UI/l)^#^	448 ± 481	102 ± 88	0.005
Creatinine (mg/dL)^#^	3 ± 1.5	2.6 ± 2	0.6
Acute graft dysfunction	5 (83%)	15 (60%)	0.38
Graft loss	0	1 (4%)	-
Death related to DI	0	1 (4%)	-
Late death not related to DI or CMV	4 (66%)	2 (8%)	0.007

*quantitative PCR (range)

^#^Reference values: Hemoglobin: 12–17 mg/dl; Leukocytes: 4,000–11,000/mm^3^; platelets: 150,000–300,000/mm^3^; Cr: 0.7–1.2 mg/dl; AST: 40 UI/l; ALT: 41 UI/l; AP: 40–130 UI/l; GGT: 1–60 UI/l.

All patients were treated as per guidelines developed for dengue infection by the Brazilian Health Ministry [22,28,29].

## Discussion

In the last few decades, the number and severity of dengue viral infection have increased substantially in endemic areas worldwide [18,30]. A global resurgence of dengue as pandemic, with emergence of severe forms in the last 25 years, has been reported, including within Brazil. [14,30–32]. During the period of January, 2007 to December, 2016, the Brazilian Health Ministry was notified of 9,131,862 DI cases in the general Brazilian population [33], and, of that number, 95,091 DI cases occurred in the city of São José do Rio Preto [34]. Approximately 75% of all DENV infections in the general population are asymptomatic, including those among adults [35].

SD occurrence may be rare in transplant recipients who have an impaired immune response [18,21]. A systematic review of literature published in 2017 found 11 publications of DI in KTx, totaling 168 cases. They suggested that physical and laboratory findings of DI in KTx do not differ from the general population, but incidence of severe dengue was significantly higher with high mortality [36].

Additionally, there have been two new publications. In one, there were 11 cases in Brazil with no long-term damage to the patients or to the grafts [37] and, in the second, there were 20 cases in India with 40% of transient renal dysfunction, and 5% of death attributed to the DI [38].

In our study, the main clinical manifestations presented in Table 2 are consistent with the literature [15,26,39–41]. Although most KTx presented similar initial symptoms to the general population with DI, severe manifestations of D+WS and SD occurred in the majority of KTx, with mortality of approximately 5%, much higher than in the general Brazilian population during this period (Brazil 2017: 0.06%; 5,434 deaths/9,131,862 DI cases), and higher than the general world population (0.1%) [33]. Hepatic impairment occurred in the majority of our patients (>60%), as mild to moderate increase in aminotransferases and GGT were observed. Severe increase in these liver enzymes was observed in two patients who had increased GGT higher than 10 times the upper limit of normal values and also had CMV infection. Combined increases of alkaline phosphatase (AP) and GGT were seen in a few patients (5/27, 26%), and no additional clinical manifestations of liver damage were noted. This is in accordance with the clinical presentation of DI in the general population and in most of the reported cases of DI in KTx [36].

The majority of DI cases reported in the literature (168 cases) had their diagnosis confirmed by IgM serology (87.5%), and contrary to the present results, only 13.7% of them were confirmed by RT-PCR/NS-1 [36]. The same pattern was observed in the last two publications, in which only one case was confirmed by RT-PCR [37,38]. In this study, 10% of the cases were confirmed by all 3 tests, 44% by 2 tests, and 18% by 1 test (NS-1 or RT-PCR). Because the city has experienced several outbreaks of DI in the last few years, and the Health Ministry guidelines don’t require that all the tests be done to confirm DI, our cases didn’t have all the possible tests performed simultaneously. RT-PCR is the gold standard, but NS1 detection was faster for patients admitted before day 5 of illness, and after that period IgM serology tests resulted in 100% positive results. Because the RT-PCR can detect up to day 15 of illness, has a low chance of cross reaction, has high specificity, and, additionally, it provides an early etiological diagnosis and the determination of current serotype [42] (both epidemiologically important and a severity predictor), we suggest always ordering RT-PCR at the same time as serologic tests in KTx, even after the usual 7 days of viremia, and even when NS1 or IgM results were negative.

Our findings corroborate others showing a higher risk of SD and death in KTx with DI [36,38,40]. Conversely, other authors have not observed any difference in the outcomes of DI in KTx and the general population [36, 37].

CMV coinfection with DI has been described in 5% of DI cases from Pakistan, but the clinical presentation was not statistically significantly different in regard to the severity of disease when compared with those who did not develop reactivation of CMV [18]. Higher prevalence (66%) of CMV reactivation was also reported [21]. CMV was always considered in the initial differential diagnosis of the cases, but the coinfection was proven in 6 of 31 cases. Interestingly, the subgroup with DI and CMV coinfection had worse thrombocytopenia, higher GGT levels, higher rate of acute graft dysfunction, and required longer hospitalization time. In spite of these conditions, graft loss and death rate related with DI were not lower compared with those that did not develop CMV coinfection. In conclusion, KTx-dengue infected patients presented initial clinical and laboratorial profiles similar to the general population. Outcomes of DI in KTx seems to have a higher risk for graft dysfunction, severe dengue, and death. The coinfection with CMV seems to aggravate clinical presentation and recovery of DI. In addition, we recommend that immunosuppression might be managed according to the clinical picture of each patient.

Our retrospective study has some limitations. First, patients were RTx that sought medical assistance and had clinical symptoms/signs suggestive of DI. Certainly, other RTx may not found it necessary to go to the hospital. Second, the tests used for diagnosis of DI were not performed simultaneously in order to provide a comparative analysis of specificity and sensibility.

Considering the RT-PCR’s ability to detect the arbovirus up to 15 days after the initial symptom, we recommend that this test should be performed even in the presence of negative NS-1 or IgM tests. CMV coinfection must be evaluated in all cases.

## Supporting information

S1 Table Dengue infection in KTx subgroup which needed immunosuppression changes compared with KTx subgroup without immunosuppression changes. (DOCX)
