# Supplementary material for: Clinical and laboratorial profiles of dengue virus infection in kidney transplant recipients: Report of a single center
Source: PLoS One. 2019 Oct 30;14(10):e0219117. doi: 10.1371/journal.pone.0219117 (PMC6821097; doi:10.1371/journal.pone.0219117)
Supplement: S1 Table — (DOCX) [file pone.0219117.s001.docx]

**S1 Table.** Dengue infection in KTx subgroup which needed immunosuppression changes compared with KTx subgroup without immunosuppression changes.

|  | **With immunosuppression changes (n=12)**  **Mean ± SD or (%)** | **Without immunosuppression changes (n=27)**  **Mean ± SD or (%)** | **p-value** |
| --- | --- | --- | --- |
| Hospitalization rate | 100% | 63% | 0.01 |
| Hospitalization time (days) | 9.8 ± 6.3 | 5.2 ± 6 | 0.008 |
| Acute renal Dysfunction rate | 92% | 44% | 0.01 |
| Nadir of serum creatinine (mg/dL) | 4.5 ± 3.9 | 2.1 ± 1.2 | 0.01 |
| Dialysis rate | 33% | 0 | 0.009 |
| CMV coinfection rate | 25% | 11% | 0.50 |
| Death rate | 17% | 0 | 0.16 |
